# Necrotizing Mucormycosis of Wounds Following Combat Injuries, Natural Disasters, Burns, and Other Trauma

**DOI:** 10.3390/jof5030057

**Published:** 2019-07-04

**Authors:** Thomas J. Walsh, Duane R. Hospenthal, Vidmantas Petraitis, Dimitrios P. Kontoyiannis

**Affiliations:** 1 Departments of Medicine, Pediatrics, and Microbiology & Immunology; Weill Cornell Medicine of Cornell University and New York Presbyterian Hospital, New York, NY 10065, USA; 2 Division of Infectious Diseases, Department of Medicine, University of Texas Health Science Center at San Antonio, San Antonio, TX 78229, USA; 3 Departments of Medicine, Weill Cornell Medicine of Cornell University, New York, NY 10065, USA; 4 Division of Internal Medicine, The University of Texas MD Anderson Cancer Center, Houston, TX 77030, USA

**Keywords:** mucormycosis, antifungal therapy

## Abstract

Necrotizing mucormycosis is a devastating complication of wounds incurred in the setting of military (combat) injuries, natural disasters, burns, or other civilian trauma. *Apophysomyces* species, *Saksenaea* species and *Lichtheimia* (formerly *Absidia)* species, although uncommon as causes of sinopulmonary mucormycosis, are relatively frequent agents of trauma-related mucormycosis. The pathogenesis of these infections likely involves a complex interaction among organism, impaired innate host defenses, and biofilms related to traumatically implanted foreign materials. Effective management depends upon timely diagnosis, thorough surgical debridement, and early initiation of antifungal therapy.

## 1. Introduction

Fungi of the order Mucorales are increasingly recognized as important causes of necrotizing wound infections in the setting of military (combat) injuries, burns, natural disaster-related, and other civilian trauma [1,2,3,4,5,6,7]. As the literature on these infections is widely distributed into journals across various disciplines, as well as lay press publications, we summarize herein the microbiology, pathogenesis, epidemiology, diagnosis, and treatment of wound-associated mucormycosis.

## 2. Microbiology

While *Rhizopus arrhizus* is the most commonly reported cause of pulmonary, sino-orbital, rhinocerebral, and disseminated mucormycosis, less commonly recognized species of Mucorales are reported worldwide in associated with trauma-related disease. *Apophysomyces* species, *Saksenaea* species and *Lichtheimia* (formerly *Absidia*) *corymbifera*, albeit uncommon causes of mucormycosis, are relatively frequently reported agents of trauma-related infections (Table 1) [4,8,9,10,11,12].

## 3. Pathogenesis

**Figure 1:**
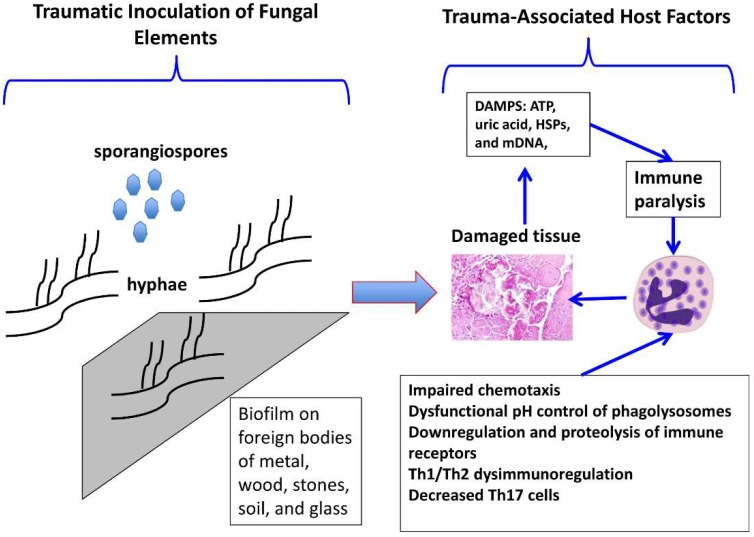
Pathogenesis of Wound Related Mucormycosis.

The pathogenesis of mucormycosis wound infections associated with trauma has not been well characterized. Unlike the cutaneous and deep soft tissue infections that occur with mucormycosis in immunocompromised patients, preponderance of patients sustaining traumatic mucormycosis in the setting of military conflict, burns, and civilian trauma, such as that associated with natural disasters and motor vehicle accidents, are immunocompetent. In the immunocompromised patient, the absence of neutrophils, or immunoregulatory dysfunction, or diabetes mellitus clearly increased the risk of locally invasive infection associated with incidental inoculation in skin and deep soft tissue. By comparison, other host and microbiological variables are likely active in the pathogenesis of infection in the immunocompetent patient. Both local and systemic immune impairment may be active in the unique settings of trauma in the previously immunocompetent patient (Figure 1). 

Leliefeld and colleagues [19] discuss in detail the impact of trauma-associated immunodysregulation and impaired function of neutrophils. Among the multiple mechanisms of trauma-related immune paralysis of neutrophils are impaired chemotaxis, dysfunctional pH control of phagolysosomes, and autocrine or paracrine serine proteolytic cleavage by neutrophil-derived serine proteases and downregulation of immune receptors (CXCR1, CXCR2, IL-2r, IL-6r, and complement receptors, including C5a). Other mechanisms associated with trauma-associated immune paralysis include downregulation of the neutrophil inflammatory response to microbial pathogens by host-tissue derived molecules with damage-associated molecular patterns (DAMPS), such as ATP, uric acid, heat shock proteins, and mitochondrial DNA, as well as release of functionally impaired circulating neutrophil populations and suppression of adaptive immunity. Gupta et al. also describe a Th1/Th2 dysimmunoregulation in patients with post-traumatic sepsis, wherein culture supernatant of T cells demonstrated elevated levels of IL-4, IL-10, and TGF-β and low levels of IL-2and IFN-γ [20]. These patients also had a T-cell immunophenotype of elevated T-regulatory cells and decreased Th17 cells. This immunodysregulation of Th1/Th2 and Treg/Th17 may further contribute to the net immunosuppression of patients sustaining trauma-associated mucormycosis. 

We hypothesize that in the setting of military injuries, civilian trauma, or wounds sustained in natural disaster, that there is a sequence of events that would include (1) direct injury to soft tissue resulting in local necrosis, as well as impaired blood flow to damaged tissue, (2) traumatic inoculation of foreign material, including soil, rocks, glass, and wood, contaminated with soil-borne Mucorales forming a nidus for possible biofilm formation, (3) establishment of local infection by the Mucorales fungus in deep tissue, and (4) possible synergistic interaction with bacteria and other fungi to establish a necrotizing infection. Since tissue has been injured, either simultaneously or previously from traumatic injury and impaired host inflammatory cells are unable to eradicate the organism, a local infection is able to be established and become self-propagating as hyphal elements further destroy local tissue, prevent capillary influx of white blood cells, and allow further proliferation of organism.

Therapeutic intervention follows from this pathogenesis in that successful management would include thorough and meticulous debridement of necrotic tissue, removal of foreign material, as well as antifungal therapy.

## 4. Epidemiology

### 4.1. Mucormycosis Following Combat-Related Injury

Combat-associated wounds complicated by invasive fungal infections (IFIs) are associated with serious morbidity and excess mortality [8]. Early observations suggested that combat wound fungal infections are more difficult to manage and had worse prognosis in comparison to non-fungal combat-related injuries [13]. In order to better understand the role of military trauma-related invasive fungal infections, particularly mucormycosis, several studies have examined the epidemiology, risk factors, outcome, and management of combat-associated mycoses, including those caused by the Mucorales. These include a series of studies of US military personnel who sustained serious injury in Afghanistan [9,21]. 

Warkentien et al. of the Infectious Disease Clinical Research Program (IDCRP) Trauma Infectious Disease Outcomes Study (TIDOS) group from the Walter Reed National Military Medical Center, Uniformed Services University of the Health Sciences, San Antonio Military Medical Center, and Landstuhl Regional Medical Center (Landstuhl, Germany) recently reviewed the impact of *Mucorales* and other invasive mould pathogens on clinical outcomes of polymicrobial traumatic wound infections [8,9]. The investigators initially reported 37 cases of IFI in combat-related wounds using a classification of proven (culture + histological evidence of angioinvasion, (*n* = 20)), probable (culture + nonvascular tissue invasion (*n* = 4)), and possible (positive fungal culture without histopathological documentation (*n* = 13)). The data were collected from records of US military personnel who served in Afghanistan. The following epidemiological and possible risk factors were common to most patients with IFI: Blast injury during foot patrol, injury occurring in southern Afghanistan, lower extremity amputation, and receipt of large volume blood transfusions [22,23]. Among the mould isolates, Mucorales were cultured in 16 cases, *Aspergillus* spp. in 16, and *Fusarium* spp. in 9. Reflecting the soil and environmental contamination of severely injured wounds, cultures yielded multiple species of moulds in 10 (28%) of these cases. The median age of the patients with IFIs was 22.9 years and 100% were male. All of the IFIs were associated with injuries sustained from blast injury. Traumatic amputations of the lower extremity above or through the knee were the most common type of initial wounds.

These investigators then compared the potential differences in microbiological features and clinical outcomes between wounds classified as IFIs (*n* = 82) and as case-matched control non-IFIs (*n* = 136). The authors further evaluated the effect of the type of mould on clinical outcome [24,25]. IFIs were predominantly secondary to fungi of the order Mucorales (35%), *Aspergillus* spp. (29%), and *Fusarium* spp. (21%). Among the 29 wound IFIs caused by Mucorales, the most common genera were *Mucor* spp. (*n* = 15 (52%), *Saksenaea vasiformis* (*n* = 5 (17%))*,* and *Rhizopus* spp (*n* = 1 (3%)). The other species of Mucorales were apparently not cultured or identified to species and may have been diagnosed histologically as mucormycosis. 

Wounds infected with a species of the Mucorales required longer median time for wound-closure in comparison to those infected with a non-Mucorales fungal pathogen (17 days vs. 13 days (*p* < 0.01)). The study also found that the median time to wound-closure was significantly longer (*p* < 0.001) for IFIs (16 days) than that for the control non-IFIs with or without skin and soft tissue infections (12 and 9 days, respectively). IFI wounds were managed principally by delayed primary closure, full-thickness skin graft, or split-thickness skin graft. Surgical amputations and revisions were more frequently performed in IFI wounds (*n* = 63 (77%)) than in non-IFI wounds (*n* = 71 (52%)). Median duration of antifungal therapy for mucormycosis was 31 days (IQR, 22–44 days). Given the broad range of fungal pathogens causing these infections, when IFI was clinically or microbiologically suspected, a combination of liposomal amphotericin B and voriconazole was empirically initiated for broad antifungal spectrum, pending definitive identification. That voriconazole is added empirically may contribute to the selection of increased virulence of isolates of Mucorales [26].

Rodriguez et al. [27] reinforced these principles of broad-spectrum combination therapy, with liposomal amphotericin B and voriconazole pending microbiological diagnosis, that was used in management of blast-related wounds suspected of having IFI during Operation Enduring Freedom in Afghanistan. Post-operative local wound management included frequent debridement of necrotic tissue, 0.025% Dakin’s solution-soaked kerlix dressing, and instillation vacuum dressings. 

### 4.2. Mucormycosis Following Natural Disasters

Mucormycosis of deep soft tissues have occurred in victims with severe injuries caused by tornadoes, hurricanes, tsunamis, and floods [10,11,12,14,15,28,29]. The organisms are inoculated following penetrating injuries from wind or water borne debris that is driven into deep soft tissues, including muscle, fascia, tendon, and bone. 

On 22 May 2011, a catastrophic EF-5 (enhanced Fujito scale; 200 MPH +) rated multiple vortex tornado devastated the community of Joplin, Missouri, USA, resulting in 13 tornado victims with serious necrotizing cutaneous mucormycosis caused by *Apophysomyces trapeziformis* following lacerations and penetrating injury from airborne material, including soil, gravel, wood, and glass [10]. *Apophysomyces trapeziformis* was definitely identified by sequencing each isolate in the D1–D2 region of the gene encoding 28S rRNA. Eleven patients suffered at least one fracture, 9 sustained blunt trauma, and 5 had penetrating injury. Multivariate analysis found that necrotizing cutaneous mucormycosis was associated with penetrating injury and increased numbers of wounds. 

Five of these patients died. *Apophysomyces* spp. are well described as causes of trauma-related musculoskeletal mucormycosis in immunocompetent hosts. However, *Apophysomyces trapeziformis* had seldom been reported as an etiological agent. Whole-genome sequencing typing (WGST), which was conducted on four isolates, demonstrated that these four isolates were separate individual strains. 

Additional WGST analysis was conducted by Etienne and colleagues from the Centers for Diseases Control (CDC) on 17 outbreak isolates and three control strains of *Apophysomyces trapeziformis*, as well as two control isolates of *Apophysomyces variabilis* [14]. While three clusters of genotypically related or identical isolates were discovered, multiple distinct isolates were also identified among the infecting organisms. The isolates from Joplin were more closely related to each other than to the control isolates, suggesting a local geographic lineage. However, there was no relation between the isolates or genotypic cluster and location within the Joplin area. Given the extensive disruption of soil by the massive tornado system, one could expect that elucidation of any such genotypic and local geographic relationship would be confounded by the wide dispersal of organisms. 

The devastating Indian Ocean tsunami of December 26, 2004, that killed more than 200,000 estimated victims with yet another estimated 40,000 seriously wounded patients [11,12,28], inflicted infectious wound complications, including trauma-associated mucormycosis, on multiple victims in Thailand, India, Sri Lanka, and other countries. These infections ranged from multifocal cutaneous mucormycosis to mucormycotic necrotizing fasciitis. Patients were described as having multiple large flap lacerations measuring as large as 60 cm in greatest diameter, particularly of the lower extremities. Among these patients, Maegele et al. reported two cases of a lethal combined infection of mucormycosis and *Fusarium* spp. *Apophysomyces elegans* was recovered from one patient. *Aspergillus fumigatus* was also recovered from one of two patients who later died. Other invasive fungal infections complicating this massive tsunami included *Cladophialophora bantiana* soft tissue infection, *Scedosporium apiospermum* brain abscess, and *Aspergillus fumigatus* brain abscess. In addition to these organisms, water-borne bacterial co-infection, including those caused by *Aeromonas hydrophila* and *Pseudomonas aeruginosa*, may have also contributed to the pathogenesis of these infections. 

Among other cases of mucormycosis associated with natural disasters, Patiño et al. reported the development of 8 cases of necrotizing soft tissue infection caused by Mucorales fungi following the cataclysmic volcanic eruption of Armero, Colombia in 1985 that resulted in more than 23,000 deaths and 4500 wounded, where burns sustained from lava, pyroclastic flows, and other fires may have allowed for inoculation from environmental pathogens [15,29]. Within the same issue, Patiño and colleagues underscore the importance of assessing necrotizing fasciitis as a syndromic clinical entity caused by many different pathogens, including Mucorales fungi. Emphasizing the importance of mucormycosis in this tragic setting, among the 38 patients with necrotizing soft tissue infection observed by Patiño and colleagues, 8 had mucormycosis. While overall mortality in patients with necrotizing fasciitis was 47.7%, it was 80% in those with mucormycosis. The authors emphasize the importance of assessing for the presence of these organisms in necrotizing soft tissue infection associated with natural disaster. 

### 4.3. Mucormycosis Following Burn Injuries

Mucormycosis of burn wounds has been known for more than one-half century to be associated with a high mortality and severe morbidity. Devauchelle et al. recently reviewed the epidemiology of mucormycosis in burn patients [5]. They identified 7 case series, 3 outbreaks and 25 case reports containing infected patients. Mortality in this review ranged from 29–100%. Kyriopoulos and colleagues from Athens, Greece reported six cases of trauma-associated mucormycosis with review of literature. Among these newly reported patients, severe thermal burns were present in 3 [16]. The other three patients suffered severe soft tissue trauma due to traffic vehicular accidents. Total body surface area of burns ranged from 45–71%. *Rhizopus* and *Rhizomucor* species were recovered in all patients. Bacterial co-infection with *Staphylococcus aureus*, *Pseudomonas aeruginosa*, *Stenotrophomonas maltophilia*, *Acinetobacter baumannii*, and *Proteus mirabilis* was identified. The authors observed that the frequency of mucormycosis in their center from 2005 to 2014 among 477 adult patients was 0.63%, which they further noted was consistent with that of Schaal et al., who reported an incidence of 0.5% in a French military burn center [17,30]. Use of contaminated bandages in the burn unit was the reason for an outbreak of *Absidia corymbifera* infection in 2005, according to Christiaens et al. [31]. Kyriopoulos et al. describe that their treatment protocol for suspected mould infections of burn wounds stipulates rapid diagnosis and extensive surgical debridement accompanied by amphotericin B in treatment of cases of mucormycosis [16]. 

### 4.4. Mucormycosis Following Civilian Industrial, Agricultural, and Automotive Injuries

In addition to military (combat) injuries, burns, and natural disasters injuries as predisposing factors for necrotizing mucormycosis, injuries associated with civilian industrial, agricultural, and automotive/vehicular accidents also pose a threat for these serious infections. Lelievre, representing the French Mycosis Study Group, published a study of posttraumatic mucormycosis [7]. Cases of posttraumatic mucormycosis were identified and reviewed from the database of the nationwide French study known as ‘‘RetroZygo” [32]. The RetroZygo study included 101 cases of proven and probable mucormycosis. Among these cases were 16 with posttraumatic mucormycosis.

Posttraumatic mucormycosis in these patients was seldom associated with underlying diseases (e.g., diabetes or malignancy) in comparison to other forms of mucormycosis. The preponderance of cutaneous mucormycosis occurred in posttraumatic mucormycosis (87%) vs. other forms of mucormycosis (7%). As these infections were localized to the skin and soft tissue and occurred in a trauma-related clinical setting, an early diagnosis was readily established. Among the causes of mucormycosis, *Apophysomyces elegans* complex and *Saksenaea vasiformis* were recovered more frequently from posttraumatic wounds than from other types of mucormycosis. More patients (94%) underwent surgery for posttraumatic mucormycosis than did those with other forms (48%). Survival at day 90 was greater in posttraumatic mucormycosis (88%) in comparison to that of other types of mucormycosis (48%).

Among the 122 cases that were identified from a systematic review of literature, traffic injuries, domestic accidents, natural disasters, and farm accidents constituted the most common events predisposing to civilian posttraumatic mucormycosis. Dissemination from traumatic mucormycosis seldom occurred (9%). *Apophysomyces elegans* complex and *Lichtheimia* (formerly *Absidia*) spp. were the two most common species recovered from these cases of civilian posttraumatic mucormycosis. *Lichtheimia corymbifera* has long been associated with post-traumatic necrotizing mucormycosis. *Apophysomyces elegans* complex was also a common organism recovered from wounds of posttraumatic mucormycosis.

### 4.5. Trauma-Related Mucormycosis in Children

Most patients reported with trauma-associated mucormycosis are adults. Little is known about trauma-associated mucormycosis in pediatric patients. Kordy and colleagues reported the development of severe deep soft tissue mucormycosis caused by *Apophysomyces elegans* in an otherwise healthy child who sustained a traumatic avulsion injury of her latissimus dorsi [18]. The traumatic inoculation occurred in Saudi Arabia in the setting of the child being thrown from an automobile during a motor vehicle accident and tearing the deep soft tissue in the soil where she had landed. The child was treated successfully with surgical debridement and systemically administered liposomal amphotericin B. This report further underscores the role of *Apophysomyces* spp. in trauma associated necrotizing mucormycosis and highlights the need for a high index of awareness of in both pediatric and adult patients.

### 4.6. Trauma-Related Osteoarticular Mucormycosis

While any of the previously mentioned settings may inflict trauma-related osteoarticular mucormycosis, the preponderance of literature addresses deep soft tissue infections. Little has been written about trauma-related osteoarticular mucormycosis. A systematic review of osteoarticular mucormycosis by the International Osteoarticular Mycoses Consortium from 1978 to 2014 [33] found that among 34 patients, seven (21%) suffered trauma as the predisposing factor. Among these 7 patients with trauma, the long bones were infected with direct inoculation as the mechanism of infection. By comparison, hematogenous dissemination is the most common mechanism in immunocompromised patients. Despite the complexity of osteoarticular mucormycosis, a combined therapeutic approach of surgical debridement and amphotericin B resulted in a favorable outcome in 82%.

## 5. Principles of Management

Strategies of management of trauma-related mucormycosis follow fundamental principles of diagnosis, empirical antifungal therapy for suspected infection, extensive surgical debridement of necrotic tissue, definitive antifungal therapy for documented disease, topical therapy, and reversal of underlying metabolic or immune-impaired conditions.

### 5.1. Microbiological Diagnosis

A heightened clinical suspicion at the time of wound assessment and a rapid laboratory diagnosis are essential in the management of trauma-related necrotizing mucormycosis [34]. Direct examination of calcofluor wet mounts of tissue samples under fluorescent microscopy may rapidly identify organisms while cultures are pending [35]. Histological sections may further confirm the presence of characteristic broad, sparsely septated, or non-septated hyphae. The presence of angioinvasion further confirms a histological diagnosis.

Deployment of PCR or other molecular diagnostic systems for laboratory diagnosis of wound-associated mucormycosis could complement conventional microbiological methods and guide pathogen-directed antifungal therapy. Rapid molecular diagnostic tools have been developed that may further aid in the diagnosis of necrotizing mucormycosis for those healthcare facilities with clinical laboratories that are resourced with dedicated assays and technologist support. Pioneering work by Kasai and colleagues developed a rapid PCR-based platform that identified several genera (*Rhizopus*, *Mucor*, *Rhizomucor*, and *Cunninghamella* species) within the Mucorales in plasma, bronchoalveolar lavage fluid, and tissue of rabbits with experimental invasive pulmonary mucormycosis [36]. The primers and probe sequences used in these assays helped in developing several subsequent PCR systems for diagnosis of mucormycosis. Millon et al. studied quantitative PCR assays detecting *Mucor*/*Rhizopus*, *Rhizomucor*, and *Lichtheimia* (formerly *Absidia)* in a retrospective multicenter study [37]. The investigators found that 36 (81%) of 44 patients had ≥ PCR-positive serum sample. The first positive PCR sample was identified in a median of 9 days before a conventional microbiological or histological diagnosis. The investigators also found that quantification of DNA loads in serum correlated with therapeutic response.

In a combined retrospective and prospective study of 77 burn victims, Legrand and colleagues identified 8 patients with wound related mucormycosis by plasma qPCR in a screening protocol of samples collected twice weekly [38]. Underscoring its utility in early diagnosis, qPCR identified the presence of wound-associated mucormycosis for a median of 11 days before a conventional diagnosis using standard microbiological or histological tools. Moreover, there was a trend toward improved survival in patients for whom pre-emptive was initiated following a molecular diagnosis of wound-associated mucormycosis.

Fréalle et al. investigated the possible role of non-sterile bandages used to secure sterile gauze and strips in contact with burn wounds in the Burn Unit of the University Hospital of Lille, France in order to determine their relationship to outbreaks of infections caused by *Lichtheimia* (formerly *Absidia)* spp. in March 2014 and July 2016, as well as in individual cases in November 2013 and July 2016. Real-time PCR, and *Lichtheimia* species-specific qPCR detected *Lichtheimia ramosa*, *Lichtheimia ornata*, and *Lichtheimia corymbifera* in crepe bandages and elasticized bandages [39]. The authors underscore the value of qPCR in molecular epidemiological investigations, the potential role of non-sterile bandages as a source of cutaneous mucormycosis in burn patients, and the need for sterile bandages in managing these wounds.

### 5.2. Surgical Management and Antifungal Therapy

As trauma-related mucormycosis is an uncommon infection, there are no controlled studies to guide management. Nonetheless, the experience from the medical command caring for servicemen with trauma-related mucormycosis provides the largest body of collective experience in management of this devastating infection [27]. The approaches outlined by Rodriguez et al. that are grounded in direct battlefield experience during Operation Enduring Freedom maintain that aggressive and frequent surgical debridement with topical antifungal therapy, such as Dakin’s solution, was the principal therapy for management of invasive fungal infections, including mucormycosis, in war wounds. When there is a strong suspicion of IFI, initial antifungal therapy consists of liposomal amphotericin B and an intravenously administered triazole, voriconazole or posaconazole. Following a diagnosis of mucormycosis, therapy was consolidated with liposomal amphotericin B.

As a guide to resection of tissue, one of the serious challenges is the need to repeatedly resect necrotic tissue resulting in larger wounds. Defining clear margins is essential in limiting resection of viable tissue while receiving infected margins. We have observed that wound margins may appear clinically and histologically intact while still having viable organisms present. We therefore have used a system of intraoperative assessment of resected tissue margins sent by the surgical team to the clinical microbiology laboratory for fluorescent microscopy using calcofluor wet mounts [40,41].

There are, of course, many variables that must be individualized for each patient. These include the extent, timing, and repeating of debridement, the duration of systemic therapy, use of oral agents, role of adjunct hyperbaric oxygen, repair of major tissue defects, and the timing of skin grafting.

## 6. Future Directions

Considerably more work is needed in understanding the pathogenesis, diagnosis and treatment of trauma associated mucormycosis. Appropriate immunocompetent animal models are paramount to understanding the pathogenesis and treatment of these infections. Understanding the environmental microbiology of trauma associated mucormycosis is important to addressing the role of non-*Rhizopus* species, such as *Lichtheimia corymbifera, Saksenaea* and *Apophysomyces* spp. Development of new rapid molecular tools, especially at point of care in a trauma setting would be highly beneficial in guiding therapy [42,43,44,45,46,47,48]. Development of new approaches for topical therapy, as well as discovery of novel antifungal agents with the potential for synergistic combinations with licensed compounds may improve therapeutic outcome, especially in eradicating residual fungi that are not removed by debridement or other surgical interventions [49,50]. Further study of the newer antifungal agents, such as posaconazole and isavuconazole, are merited [51]. Finally, the potential for novel tissue regenerative systems offers potential new approaches in management of the wounds associated with trauma-related mucormycosis.

## Figures and Tables

**Table 1:** Necrotizing Mucormycosis involving Wounds.

Reference	Cause of Injury	Number of Patients	Organisms Recovered (*n*)	Location
Warkentien et al. 2015 [8]	Combat-related injury	29	*Mucor* spp. (15), *Saksenaea vasiformis* (5), *Rhizopus* spp. (1)	Afghanistan
Paolino et al. 2012 [13]	Combat-related injury	2	*Mucor* sp. (1), *Absidia* sp. (1)	Afghanistan
Warkentien et al. 2012 [9]	Combat-related injury	16	*Mucor* spp. (9), *Saksenaea vasiformis* (6), *Apophysomyces* spp. (2)	Afghanistan
Neblett Fanfair et al. 2011 [10]	Tornado, 2011	13	*Apophysomyces trapeziformis*	Joplin, Missouri
Maegele et al. 2006 [11]	Tsunami, 2004	1	*Apophysomyces elegans*	Southeast Asia
Andresen et al. 2005 [14]	Tsunami, 2004	1	*Apophysomyces elegans*	Sri Lanka
Snell et al. 2007 [12]	Tsunami, 2004	1	*Apophysomyces elegans*	Thailand
Kyriopoulos et al. 2015 [15]	Burn injuries and soft tissue automotive injury	6	*Rhizopus* spp. (3), *Rhizomucor* spp. (3)	Greece
Schaal et al. 2015 [16]	Burn injuries	9	NS	France
Christiaens et al. 2005 [17]	Burn injuries	7	*Absidia* (currently *Lichtheimia) corymbifera*	Belgium
Lelievre et al. 2014 [7]	Civilian industrial, agricultural, and automotive injuries	16	*Apophysomyces elegans* complex, *Saksenaea vasiformis*	France
Kordy et al. 2004 [18]	Automotive injury	1	*Apophysomyces elegans*	Saudi Arabia

NS: not specified.
